# Clarifying workforce flexibility from a division of labor perspective: a mixed methods study of an emergency department team

**DOI:** 10.1186/s12960-020-0460-7

**Published:** 2020-03-06

**Authors:** Sarah Wise, Christine Duffield, Margaret Fry, Michael Roche

**Affiliations:** 1 Centre for Health Economics Research and Evaluation, University of Technology Sydney, PO Box 123, Broadway, NSW 2007 Australia; 2 Faculty of Health, University of Technology Sydney, PO Box 123, Broadway, NSW 2007 Australia; 3 School of Nursing and Midwifery, Edith Cowan University, Australia, 270 Joondalup Drive, Joondalup, WA 6027 Australia; 4 Director Research and Practice Development Nursing and Midwifery Directorate, Northern Sydney Local Health District, Royal North Shore Hospital, Kolling Building, St Leonards, NSW 2065 Australia

**Keywords:** Workforce flexibility, Functional flexibility, Division of labor, Mixed methods research, Time study, Emergency department, Healthcare workforce, Workforce reform

## Abstract

**Background:**

The need for greater flexibility is often used to justify reforms that redistribute tasks through the workforce. However, “flexibility” is never defined or empirically examined. This study explores the nature of flexibility in a team of emergency doctors, nurse practitioners (NPs), and registered nurses (RNs), with the aim of clarifying the concept of workforce flexibility. Taking a holistic perspective on the team’s division of labor, it measures task distribution to establish the extent of multiskilling and role overlap, and explores the behaviors and organizational conditions that drive flexibly.

**Methods:**

The explanatory sequential mixed methods study was set in the Fast Track area of a metropolitan emergency department (ED) in Sydney, Australia. In phase 1, an observational time study measured the tasks undertaken by each role (151 h), compared as a proportion of time (Kruskal Wallis, Mann-Whitney *U*), and frequency (Pearson chi-square). The time study was augmented with qualitative field notes. In phase 2, 19 semi-structured interviews sought to explain the phase 1 observations and were analyzed thematically.

**Results:**

The roles were occupationally specialized: “Assessment and Diagnosis” tasks consumed the largest proportion of doctors’ (51.1%) and NPs’ (38.1%) time, and “Organization of Care” tasks for RNs (27.6%). However, all three roles were also multiskilled, which created an overlap in the tasks they performed. The team used this role overlap to work flexibly in response to patients’ needs and adapt to changing demands. Flexibility was driven by the urgent and unpredictable workload in the ED and enabled by the stability provided by a core group of experienced doctors and nurses.

**Conclusion:**

Not every healthcare team requires the type of flexibility found in this study since that was shaped by patient needs and the specific organizational conditions of the ED. The roles, tasks, and teamwork that a team requires to “be flexible” (i.e., responsive and adaptable) are highly context dependent. Workforce flexibility therefore cannot be defined as a particular type of reform or role; rather, it should be understood as the capacity of a team to respond and adapt to patients’ needs within its organizational context. The study’s findings suggest that solutions for a more flexible workforce may lay in the organization of healthcare work.

## Background

Many governments are concerned that the current healthcare workforce is not flexible enough to meet increasing and more complex health demands or to achieve efficiency of scarce resources [1–3]. In the search for a more flexible workforce, reforms have created new roles and altered existing roles by redistributing tasks through the workforce [4]. While “flexibility” is often used as a rationale for such reforms, it is never defined as an outcome [5, 6].

It is often claimed that the healthcare workforce is inflexible because of the way tasks are bound to particular occupations and that this occupational specialization has perpetuated the inefficient use of healthcare skills [4, 7]. That certain occupations perform certain tasks with an associated set of specialist knowledge and skills is a characteristic of the organization of work in every industrialized society, and is described as the “division of labor” [8, 9]. At the workplace level, the division of labor may be understood from two epistemological positions: first, by measuring the distribution of the organization’s tasks between occupational roles within a team; second, by understanding the social relationships that organize and coordinate the work of those occupations [10]. The second, constructivist perspective has dominated the analysis of the healthcare division of labor, particularly in the seminal works of Freidson [11], Larson [12], and Abbott [13] that sought to explain the dominant status the medical profession has achieved by controlling diagnosis and treatment tasks. Some of the most significant and widespread workforce reforms have redistributed these tasks to nurses. While there is an abundance of literature charting and evaluating such reforms [14, 15], there has been no examination of whether they have resulted in a more flexible workforce.

This study used the concept of “functional flexibility” to identify whether the division of labor within a team of emergency doctors and nurses can be described as “flexible.” Functional flexibility refers to an organization’s ability to deploy the skills of its employees to respond and adapt to changes in workload, processes, or technology [16, 17]. It is achieved across three dimensions of work organization: (i) increasing the range of skills workers possess and the tasks they undertake (multiskilling), (ii) using teamwork rather than managers to coordinate work, and (iii) enhancing levels of worker autonomy [16, 17]. The original study on which this paper is based examined functional flexibility across all three dimensions [6, 18]. This paper focusses on the first dimension: multiskilling.

Under functional flexibility, workers are multiskilled for greater responsiveness and adaptability to fluctuations in demand. Compared with narrowly specialized workers, multiskilled workers can complete more tasks within a whole work process to reduce transaction costs (e.g., communication, errors, and delays caused by the delegation of tasks between specialist workers) and idle time (i.e., when a specialist worker has no tasks to complete) [19]. In reality, most teams comprise specialist roles but are sufficiently multiskilled for there to be an overlap in the tasks they can perform [20]. Using this role overlap to share tasks in response to changing workload demands is central to the responsiveness of multiskilling [16, 21].

No previous studies have directly examined the functional flexibility within a team of doctors and nurses. Indeed, despite much theorizing on the doctor-nurse division of labor, surprisingly, little is known about precisely who does what in healthcare teams [22, 23]. As Larkin [24] observed 35 years ago, healthcare workforce reforms tend to be based on “… a call for ‘flexibility’ and ‘teamwork’ … rather than any systematic analysis of the tensions of the resulting division and redivision of labor.” This study seeks to address these gaps. It aims to define the concept of workforce flexibility in the healthcare context by taking a holistic perspective on the division of labor within an Australian emergency department (ED) team: the distribution of tasks and the social relationships between clinicians.

## Methods

The two epistemological components of the division of labor adopted as the lens for the study necessitated a mixed methods research design. An explanatory sequential mixed methods design employed work observations (quantitative time study and qualitative field notes) to measure the task distribution between doctors, nurse practitioners (NPs), and registered nurses (RNs) (referred to collectively as “clinicians”). This was followed by qualitative interviews to explore the social relationships and organizational context that *explain* that distribution [25]. In doing so, the study also addresses a criticism that mixed methods studies in healthcare often lack an explicit theoretical foundation [26, 27].

### Study site

Emergency departments are at the forefront of reforms that have redistributed medical tasks to nurses, potentially creating a large overlap in the tasks that doctors and nurses perform. Registered nurses can prescribe medications to manage patients’ symptoms (e.g., pain and anti-nausea medications) and order diagnostic investigations (e.g., X-ray and pathology) to improve the timeliness and quality of patient care [28]. The nurse practitioner (NP) role has also been widely adopted in EDs to autonomously diagnose and treat patients with less complex conditions [29]. The ED was therefore selected as a possible exemplar of functional flexibility within a healthcare team.

The study site was the ED of a metropolitan tertiary referral hospital in Sydney, Australia. The department was typical of other EDs of its type in terms of the range of patients treated, models of care, and clinical roles [30]. There were 146 full-time equivalent (FTE) nurses (including four NPs) and 64.5 FTE doctors of whom 28.5 FTE were junior doctors on rotation through the hospital. The study was set in the “Fast Track” area of the ED, dedicated to quickly treating and discharging patients with minor injuries and less complex conditions, since this is where the NPs predominantly worked.

### Mixed methods study design

**Fig. 1:**
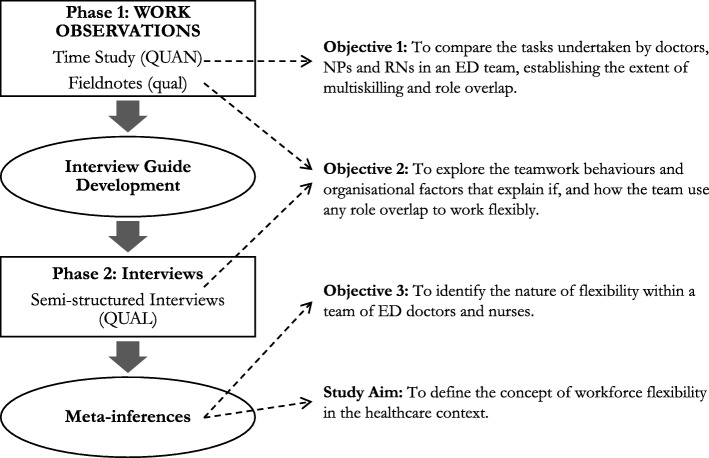
Explanatory sequential mixed methods study design

The study’s design linking the research objectives with appropriate quantitative and qualitative methods is provided in Fig. 1. Following the guidelines for mixed methods, diagrams recommended by Ivanakova et al. [31], the rectangular boxes indicate a data collection phase, the ovals represent a point where qualitative and quantitative data are integrated, and the solid arrows indicate the sequencing.

### Phase 1: Work observations: time study and field notes

Over a period of 3 months, clinicians were observed as they conducted their work in the Fast Track using the quantitative method of time study and the qualitative method of field notes. Time study involves breaking down work processes into mutually exclusive task categories and recording the time the staff spend performing those tasks. Time study data were collected on a tablet running the “Work Observation Method by Activity Timing” (WOMBAT) software [32]. There were 21 task categories, aggregated under six top-level categories for the analysis (Table 1). The development of these categories is described in Additional file 1. Details for some types of tasks were recorded in a separate spreadsheet, for example, the type of test or procedure observed (e.g., performing venipuncture or ordering X-ray). These data are referred to as the “task details data.”

**Table 1:** Task categories used for data analysis

**Assessment and Diagnosis**	**Tests and Procedures**	**Medication**
Patient assessment Vital signs (blood pressure, temperature, etc.) Diagnosis—checking results and consulting colleagues Supervision (supervisor/supervisee) Documentation	Order tests and procedures Prepare tests and procedures Perform tests and procedures	Prescribe medications Administer medications Discuss medications
**Patient Communication and Comfort**	**Organization of Care**	**Off task**
Patient communication Patient comfort—food or water, physical comfort, hygiene needs, and escorting	Electronic waiting list Professional communication Unit administration Tidy—maintaining the care environment	Locate—notes, patients, forms, and colleagues In transit Social Waiting

The time study sample size is determined by the frequency of tasks observed and articulated as the number of observation hours. An estimated meaningful difference of 50% in the amount of time each role spent performing tasks in the top-level task categories was used to calculate the sample size. Based on this medium effect size, a power calculation indicated that a minimum sample size of 134 observation hours was required (two-tailed, Wilcoxon-Mann-Whitney test with a significance of 0.05 and a power of 0.8; GPower 3.1). Table 2 shows a total of 152.7 h of observation data were collected over 104 observation sessions: 32 observation sessions were undertaken with doctors (DR), 34 with NPs, and 38 with RNs. All four NPs employed by the ED participated in the study and were observed multiple times. Eight RNs and six doctors were observed twice.

**Table 2:** Time study sample

	DR	NP	RN	Total
Total observations (h)	49.5	51.6	51.6	152.7
Mean session time (min)	95	92	82	N/A
Number of sessions	32	34	38	104
Number of tasks recorded	1594	1665	2700	5959

A purposive sampling strategy was adopted to enroll time study participants. Only clinicians with a patient load were included. This excluded emergency physicians (the most senior doctors) and the nurse coordinator (a senior RN) who performed consultation and oversight functions. A short demographic questionnaire was administered before each observation session. The researcher then shadowed one participant at a time from a discrete distance, coding all the tasks they performed into the time study software. Only one researcher conducted the time study observations to maintain consistency in task coding. The data were analyzed using IBM SPSS Statistics v22. Task frequency is presented as the number and proportion of the count of all tasks observed, and the difference between the three roles tested with Pearson’s chi-square. Task time is presented as a proportion of total task time and the difference between roles tested using Mann-Whitney (pair-wise) and Kruskal-Wallis (overall).

The qualitative observation method of field notes captured those aspects of the team’s division of labor that could not be measured quantitatively, especially teamwork behaviors and the organizational context (e.g., patient demand and staffing arrangements) that explain if and how the team worked flexibly. Field note data were managed with NVivo v11 and analyzed using the deductive, template method [33]. The time study task categories were used as a priori themes for initial coding (e.g., field note entries related to the prescription of medication were coded together) [26, 34]. The analysis then proceeded inductively, identifying themes that cut across, or were not captured by the quantitative task categories [26, 33]. This analysis integrated the quantitative and qualitative data within phase 1 and informed the development of the interview guide for phase 2 [35].

### Phase 2: Qualitative interviews

In phase 2, 19 semi-structured interviews (RN = 8, DR = 7, NP = 4) sought to explain the phase 1 observations and provided insights into the division of labor not accessible from observation alone, such as clinicians’ decision-making criteria. The sample included senior doctors and nurse coordinators excluded from the phase 1 time study.

Interview transcripts were managed with NVivo v11 and analyzed thematically [36]. In the first stage of analysis, open codes were created by noting frequently recurring and evocative phrases, ideas, and perceptions [37]. This data-driven, inductive approach was complemented by a deductive approach, using themes derived from the phase 1 findings and the research objectives. This balanced inductive and deductive approach achieved coherence and theoretical rigor across the study while allowing new interpretations to arise from the data [38]. A conformability audit was conducted by a second researcher [39].

### Meta-inferences

The study’s meta-inferences were drawn using a cross-over mixed analysis [40]. The findings were synthesized into a coherent whole by combining the quantitative tables of results and associated field note evidence from phase 1 and reported interview themes from phase 2 into a single NVivo project. The whole dataset was then analyzed thematically using the balanced inductive and deductive method described above.

## Results

The results of phase 1 and phase 2 are presented using the narrative weaving approach [41], merging the quantitative and qualitative findings and discussing them together as three themes that describe the nature of flexibility in the ED team: specialized multiskilled roles, the flexibility of overlapping roles, and the organizational conditions for flexibility.

### Specialized multiskilled roles

The time study data show the three roles were both occupationally specialized, as indicated by the task categories that consumed the highest proportion of their time, and multiskilled, undertaking tasks across all categories. The key results from the phase 1 time study are given in Table 3. The upper section of the table gives the mean proportion of time each role spent performing tasks in that category as a proportion of all task time. The lower section reports the results for the non-parametric tests of significant differences between the three roles, pairwise, and overall. Significant probability values at *P* < .05 are in bold font.

**Table 3:** Mean proportion of time on top-level task categories, with pairwise and overall significance

	Assessment and Diagnosis	Investigations and Procedures	Medication	Organization of Care	Patient Communication and Comfort	Off task
DR*	51.1% (*12.4*)	9.7% (*12.7*)	7.2% (*5.9*)	12.2% (*6.4*)	9.2% (*4.4*)	10.5% (*11.6*)
NP*	38.1% (*15.8*)	14.9% (*12.1*)	6.9% (*8.8*)	14.6% (*10.4*)	14.3% (*8.6*)	11.3% (*10.8*)
RN*	22.4% (*11.3*)	13.6% (*10.6*)	14.4% (*11.4*)	27.6% (*13.5*)	8.1% (*4.7*)	14.0% (*15.8*)
DR/NP**	≤ .001 (*276.0*)	.078 (*406.5*)	.342 (*470.0*)	.546 (*497.0*)	.013 (*350.0*)	.617 (*505.0*)
DR/RN**	≤ .001 (*67.0*)	.061 (*449.5*)	.012 (*394.0*)	≤ .001 (*132.0*)	.203 (*500.0*)	.437 (*542.0*)
NP/RN**	≤ .001 (*280.0*)	.623 (*602.5*)	.002 (*374.5*)	≤ .001 (*230.0*)	≤ .001 (*355.0*)	.778 (*621.0*)
Overall***	≤ .001 (*46.6*)	.108 (*4.6*)	.003 (*11.3*)	≤ .001 (*36.8*)	.002 (*12.5*)	.732 (*0.6*)

Significant results bolded at *P* < .05

*Mean total time on task, % (*SD*)

**Mann-Whitney *U*, *p* (*U*)

***Kruskal Wallis, *p* (*χ*^2^)

Assessment and Diagnosis tasks consumed the majority of doctors’ and NPs’ time. This reflects both roles’ primary function to determine the diagnosis, treatment, and disposition of Fast Track patients. While sharing this role function, doctors and NPs were differentiated into two key ways. First, NPs spent a significantly higher proportion of their time on Patient Communication and Comfort tasks than doctors (14.3% vs. 9.2%, *U =* 350, *P* = .013). The observations recorded in the field notes show NPs provided patient education to support ongoing care, combining the focal medical tasks of diagnosis and treatment with the core nursing values of holistic, patient-centered care. Second, doctors and NPs were differentiated by the range of patient conditions they treated. Nurse practitioners’ specialization in minor injuries, such as wounds and fractures, was not exclusive, but their focus on these patients was evident across the data. For example, NPs spent a significantly higher proportion of their time preparing and performing procedures (a subcategory of “Investigations and Procedures”) (13% compared to 7.8%, *U =* 386, *P* = .041) than did doctors (Table 4, Additional file 2). Further, the task details data show NPs performed 43 out of the 69 treatment-related procedures observed, for example, wound repair and musculoskeletal treatments (Table 5, Additional file 2). Doctors performed just nine of the treatment-related procedures observed.

The Organization of Care category consumed the highest proportion of RNs’ time. Within this task category, Professional Communication, the giving and receiving of instructions and information about patient care, accounted for 16.2% of RNs’ time, significantly higher than for doctors (9.0%, *U =* 222, *P* ≤ .001) and NPs (7.8%, *U =* 239, *P* ≤ .001) (Table 6, Additional file 2). Registered nurses also spent a significantly higher proportion of their time on medication tasks. In particular, RNs performed 82% (*n* = 255) of all the medication administration tasks observed while NPs performed 14% (*n* = 44) and doctors only 4% (*n* = 13, *χ*^2^ = 181.7, *P* ≤ .001) (Table 7, Additional file 2).

Each roles’ specialization was evident in the data, but it was also clear that they were multiskilled. Other than medication administration, there were few tasks recorded in the time study which were exclusively, or even predominantly, performed by one role. This created an overlap between the three roles which they used to work flexibly in response to patients’ needs.

### The flexibility of overlapping roles

There were two forms of role overlap within the team. The first occurred where RNs undertook the traditional medical tasks of diagnosis and treatment. In the Fast Track model of care, RNs were the first to clinically assess the patient to identify any investigations to be ordered and symptoms to be managed. They were also responsible for the ongoing monitoring of waiting patients. These activities meant RNs spent 22.4% of their time on Assessment and Diagnosis tasks (Table 3). Registered nurses were also observed to prescribe medications 26 times (23% of all “prescribing” tasks) (Table 7, Additional file 2) and order investigations on 24 occasions (23% of the “order investigation” tasks) (Table 8, Additional file 2). Thus, while responsibility for a formal diagnosis decision rested with a doctor or NP, this process was shared with RNs to improve the timeliness and responsiveness of patient care. Indeed, it was observed in phase 1 and confirmed by the interview data that the medications and investigations ordered by RNs were sometimes all Fast Track patients needed to diagnose and treat their condition, as this doctor explains:

If the nurses have taken the initiative and done bloods, they’ve got the urine, they’ve prescribed some pain medication. By the time I go and [assess the patient], they feel a lot whole better. The bloods are normal. I literally can see [the patient] very quickly and say “Everything is back. Everything is fine. You’re fine” Interview - DR7

In the second form of role overlap, the team shared everyday clinical tasks, particularly “Investigations and Procedures” where there was no significant difference between the three roles (Table 4). The task details data show that venipuncture was the most commonly observed shared task (*n* = 36) (Table 9, Additional file 2). Registered nurses performed more venipuncture procedures than any other role (*n* = 17) since they could initiate this task themselves or have it delegated from a doctor or NP. Interactions between team members recorded in the field notes indicated that doctors and NPs sometimes chose to perform a venipuncture procedure themselves and sometimes delegated that procedure to an RN.

A doctor was looking for someone to take a patient’s blood, they asked an RN “who’s looking after the beds?” The RN pointed to the nurse who was very busy at that moment. When the doctor saw how busy the nurse was they said, “That’s OK I'll do it myself.” Fieldnote - 2/4/15

In the interviews, doctors and NPs consistently reported two key factors that guided their decision whether to delegate a task or to perform it themselves. The first was the need to respond to a patient’s need based on how urgent or technically difficult the task, as this senior doctor explains:

For example, if someone needs venous access straight away because they’re critically ill then I’ll do it myself… So definitely, there’d be reasons of expediency where I want to do things myself. Interview - DR6

The second factor was their workload relative to that of the RNs. Doctors and NPs would perform the task themselves if the RNs did not have the capacity to complete the task within an appropriate timeframe, particularly when the RNs were overwhelmed with tasks which were part of their specialist role.

… there are things that the nurses do that we don’t do and that should be their priority. So if they’re busy getting admission papers sorted so that a patient can go to the ward and it means that my cannula is going be delayed whilst they’re sorting that out, well, it makes more sense for me to do it myself. Interview - NP3

From the other side of the delegation process, interview data reveal a willingness among RNs to perform a delegated shared task when the doctors and NPs were overwhelmed with *their* specialist role of assessing and diagnosing patients. Evaluating relative workloads within the delegation process sought to prevent the overloading of a particular role, to keep patients flowing through the ED. The quote below highlights a sentiment that consistently ran through the interview data that “being flexible” in the ED meant doctors and nurses using their overlapping roles to share tasks in response to workload demands:

… the doctors also do nursing stuff, they’ll go and do a [urinary analysis] or a set of obs [vital signs] on the patient to get stuff done, but then you get doctors who are like, “No. I’m not doing that,” so the nurses will say “Well I’m not doing your job”. Things like that can happen and that’s where it doesn’t work … and other people are more flexible, willing to work together and help each other out. Whatever needs to be done, just do it. No matter if you’re a doctor or a nurse, just get it done. Interview - RN8

However, as interviewee RN8’s comment highlights, not all team members demonstrated this flexible behavior, and conflicts over task responsibility could arise causing delays and task duplication.

### Organizational conditions for flexibility

The team’s ability to use their overlapping roles to work flexibly rested on the experience of its core, permanent staff and their deep understating of ED roles, processes, and workload. Interviewees noted that those new to the department, especially junior doctors on rotation, did not possess this knowledge. In contrast, experienced ED clinicians had a shared understanding of how to respond to workload demands, could anticipate what other team members needed to complete their tasks, and use their overlapping roles to share tasks when required, as this senior doctor explains:

Well, I think the more senior the person is, both medical and nursing, I suspect they work better in terms of … trying to help each other rather than working independently, or in effect duplicating the work. That would be one aspect, the experience of knowing or anticipating what the other person can do. Interview - DR3

There were organizational conditions specific to the ED that allowed doctors and nurses to develop this knowledge. In other areas of the health system, senior doctors work across many different teams. In the ED, senior doctors, NPs, and RNs were permanently based in the department; they shared a workspace and often executed tasks concurrently allowing them to develop a deep understanding of each other, how ED workload impacts each role, and their interdependency. These organizational conditions, combined with the urgency and unpredictability of ED work, explain why the team was willing to use their overlapping roles to work flexibly.

## Discussion

By taking a holistic division of labor perspective, the study has, for the first time, described the task distribution within a healthcare team in sufficient detail to empirically demonstrate that nursing and medical roles can be both occupationally specialized and multiskilled, with considerable overlap in the tasks they perform. Observations of, and clinicians’ perspectives on, the behavior and organizational context that shaped their division of labor revealed that clinicians used their multiskilled, overlapping roles to work flexibly: sharing tasks to respond to patients’ needs and to adapt to changing workload demands, consistent with the responsiveness of functional flexibility [16, 21]. Unlike the typical characterization of healthcare teams reported in the literature [42, 43], but typical of other ED teams [44], the team understood their roles were interdependent and worked towards a shared goal of timely, quality patient care. This teamwork behavior was necessitated by the urgency and unpredictability of ED work and facilitated by the permanent ED clinical staff who worked side-by-side to develop a deep knowledge of each other’s roles. Ironically, what allowed the team to work flexibly was the stability provided by a core group of experienced ED doctors and nurses. These findings are in line with studies in other healthcare settings where there is a growing recognition of a positive relationship between team experience, team member familiarity, and the team’s ability to efficiently coordinate their tasks to deliver safe patient care [45–47].

Not every healthcare team requires the functional flexibility found in the ED team (i.e., extensive task sharing between multiskilled professionals) since that was shaped by patient needs and the organizational context of emergency work. Indeed, the study’s confinement to the Fast Track area of a metropolitan ED, means the nature of the flexibility found in the team may not resonate with that of other ED treatment areas, or smaller or geographically different EDs. However, the purpose of looking in-depth at the work of one team was not to present a model of flexibility suitable for every healthcare workplace; rather, it demonstrates that a division of labor perspective can elucidate the nature and drivers of the workforce flexibility within a team. The roles, tasks, and teamwork behaviors a team requires to “be flexible” (i.e., responsive and adaptable) are highly context dependent. Future research could use the method described here to identify the nature of flexibility in other contexts, such as hospital wards, geographically dispersed teams, and primary care. The study was also limited to the roles that delivered patient care within the Fast Track area of the ED and did not include the work of other occupational groups such as paramedics, ancillary and administrative workers, and allied health professionals. Future research could include more occupationally diverse teams. This evidence would refine and deepen our understanding of workforce flexibility in the healthcare context.

The study’s holistic division of labor perspective on healthcare work has implications for other areas of health workforce research. Across disciplines, researchers and policy-makers tend to focus on only one aspect of the division of labor. Evaluations of workforce reforms are primarily concerned with quantifying the safety and quality outcomes for a specific role [48] or the extent of task redistribution [15] without understanding the impact of reforms on the whole, multi-professional team in its workplace context [49, 50]. Likewise, teamwork research has focused on the social relationships needed for good teamwork in isolation from teams’ tasks and the context in which they work [22, 23]. Salas et al. [51] argue that this narrow understanding of teamwork behavior has contributed to a lack of sustained improvement from teamwork interventions. Finally, the study’s method can potentially fill a gap in the quantitative and qualitative data required for needs-based, multi-professional workforce planning [52, 53]. Though labor-intensive, work observations comprising time study and field notes generate robust data on human resource utilization within models of care, and its effectiveness and efficiency in meeting patient needs, data that workforce planning models presently lack.

## Conclusion

The slow pace of reform towards more responsive, adaptable, and efficient healthcare services is often blamed on the occupationally controlled division of labor and the inflexibility of the professions within it [4, 7]. This study has revealed that the view of the healthcare professions as inflexible, specialized occupations must be balanced with an understanding of the inherent flexibility of multiskilled nursing and medical roles, and the increasing overlap in the tasks they perform. Lessons from the ED suggest encouraging the use of this potential flexibility to deliver more responsive care may lay in the organization of work to promote greater stability within healthcare teams [23].

By elucidating the precise nature of and drivers of flexibility in the ED team, the study has helped clarify the concept of workforce flexibility for the broader healthcare context. We conclude that workforce flexibility is not a particular type of reform or role or an outcome in itself. Rather, workforce flexibility should be understood as the division of labor (the roles, tasks, and behavior) that allows a team to respond and adapt to patients’ needs within its organizational context.

## Supplementary information

**Additional file 1.** Development of task categories for data analysis.

**Additional file 2.** Supplementary tables.

## Data Availability

Ethics approval for the study precludes the publication of the dataset. All results reported in the paper are provided in tables.

## Abbreviations

DR: Doctor
ED: Emergency department
FTE: Full-time equivalent
NP: Nurse practitioner
RN: Registered nurse
