# Supplementary material for: Clarifying workforce flexibility from a division of labor perspective: a mixed methods study of an emergency department team
Source: Hum Resour Health. 2020 Mar 6;18:17. doi: 10.1186/s12960-020-0460-7 (PMC7060538; doi:10.1186/s12960-020-0460-7)
Supplement: Supplementary file 1 — Additional file 1. Development of task categories for data analysis. [file 12960_2020_460_MOESM1_ESM.docx]

**Development of task categories for data analysis**

The task categories used for data collection in the time study were similar to those in other studies using the WOMBAT software, and are described below.

**Task categories used for data collection**

| **Direct Care** | **INDIRECT CARE** | **Medication** |
| --- | --- | --- |
| *Patient assessment*  *Vital signs (blood pressure, temperature etc.)*  *Perform tests and procedures*  *Patient communication*  *Patient comfort - food or water, physical comfort, hygiene needs, escorting*  *Supervision (Supervisor/Supervisee)* | *Order tests and procedures*  *Prepare tests and procedures*  *Locate - notes, patients, forms, colleagues*  *Diagnosis - checking results & consulting colleagues*  *Electronic waiting list*  *Tidy – maintaining the care environment* | *Prescribe medications*  *Administer medications*  *Discuss medications* |
| **DOCUMENTATION** | **Professional Communication** | **UNIT ADMINISTRATION** |
| **WAITING** | **SOCIAL** | **IN TRANSIT** |

These categories follow the health services research norm of categorising clinical tasks as ‘direct’ or ‘indirect’ care and reflect efficiency concerns about the amount of time clinicians spent on patient care tasks (direct and indirect) relative to other tasks. The current study has a greater focus on the nature of the tasks than previous WOMBAT studies, and this detail was obscured by the direct/indirect care categorisation. For example, splitting the whole process of undertaking a procedure between Direct Care (performing the procedure with the patient) and Indirect Care (preparing for, and cleaning up after the procedure) does not measure the total time that procedures consume. To overcome this problem, tasks were regrouped into new top-level categories that better reflected the nature of the tasks performed (Table 1). Regrouping existing tasks into new top-level task categories, rather than recoding tasks means the definitions of the tasks remained the same, maintaining the integrity of the data collected.
