# Supplementary material for: Clarifying workforce flexibility from a division of labor perspective: a mixed methods study of an emergency department team
Source: Hum Resour Health. 2020 Mar 6;18:17. doi: 10.1186/s12960-020-0460-7 (PMC7060538; doi:10.1186/s12960-020-0460-7)
Supplement: Supplementary file 2 — Additional file 2. Supplementary tables. [file 12960_2020_460_MOESM2_ESM.docx]

**Table 4 Mean proportion of time on Investigations and Procedures tasks with pairwise and overall significance**

|  | **Order** | **Prepare/Perform** | **Total** |
| --- | --- | --- | --- |
| DR^*^ | 1.9%  (*2.1*) | 7.8%  (*12.2*) | 9.7%  (*12.7*) |
| NP^*^ | 1.8%  (*3.0*) | 13.0%  (*12.2*) | 14.9%  (*12.1*) |
| RN^*^ | 1.4%  (*2.5*) | 12.2%  (*10.6*) | 13.6%  (*10.6*) |
| DR/NP ** | .427  (*484*.0) | **.041**  (*386.0*) | .078  (*406.5*) |
| DR/RN ** | .055  (*452.0*) | **.022**  (*416.0*) | .061  (*449.5*) |
| NP/RN ** | .405  (*577.0*) | .865  (*631.0*) | .623  (*602.5*) |
| Overall*** | .186  (*3.4*) | **.046**  (*6.2*) | .108  (*4.5*) |

* Mean proportion of total time on task - % (*SD*)

** Mann-Whitney U - p (*U*)

*** Kruskal Wallis - p (*χ^2^*)

Significant results bolded at p<.05

**Table 5 Count of treatment-related procedures performed, recorded in task details spreadsheet**

|  | DR  n | NP  n | RN  n | **Total**  **n** |
| --- | --- | --- | --- | --- |
| Wounds | 4 | 25 | 13 | **42** |
| Musculoskeletal | 2 | 14 | 3 | **19** |
| Surgical | 3 | 1 |  | **4** |
| Removal of ring (using cutter) |  | 3 | 1 | **4** |
| **Total** | **9** | **43** | **17** | **69** |

**Table 6 Mean proportion of time on Organisation of Care tasks with pairwise and overall significance**

|  | **EWL^†^** | **Professional Communication** | **Tidying** | **Unit Administration** | **Total** |
| --- | --- | --- | --- | --- | --- |
| DR^*^ | 2.6%  (*5.3*) | 9.0%  (*2.3*) | 0.2%  (*0.5*) | 0.8%  (*1.2*) | 12.2%  (*6.4*) |
| NP^*^ | 2.4%  (*5.7*) | 7.8%  (*3.5*) | 0.3%  (*0.8*) | 4.1%  (*7.4*) | 14.6%  (*10.4*) |
| RN^*^ | 7.1%  (*8.3*) | 16.0%  (*7.0*) | 1.0%  (*2.9*) | 3.3%  (*7.5*) | 27.6%  (*13.5*) |
| DR/NP ** | .173  (*438.5*) | .121  (*409.0*) | .490  (*513.5*) | **.003**  (*318.0*) | .546  (*497.0*) |
| DR/RN ** | **≤.001**  (*330.5*) | **≤.001**  (*222.0*) | **.004**  (*419.0*) | **.002**  (*354.0*) | **≤.001**  (*132.0*) |
| NP/RN ** | **≤.001**  (*313.5*) | **≤.001**  (*239.0*) | **.020**  (*480.0*) | .777  (*621.0*) | **≤.001**  (*230.0*) |
| Overall*** | **≤.001**  (*18.1*) | **≤.001**  (*28.9*) | **≤.001**  (*10.8*) | **.004**  (*11.9*) | **.003**  (*36.8*) |

* Mean proportion of total time on task - % (*SD*)

** Mann-Whitney U - p (*U*)

*** Kruskal Wallis - p (*χ^2^*)

^†^Electronic Waiting List Significant results bolded at p<.05

**Table 7 Frequency of ‘Medication’ tasks observed by role**

|  | **DR**  **n (%)** | **NP**  **n (%)** | **RN**  **n (%)** | **Total**  **n** | **p (*χ^2^*)** |
| --- | --- | --- | --- | --- | --- |
| Prescribe medications | 53  (47%) | 34  (30%) | 26  (23%) | **113** | **≤.001**  (*30.3*) |
| Administer medications | 13  (4%) | 44  (14%) | 255  (82%) | **312** | **≤.001**  (*181.7*) |
| Discuss medications | 141  (30%) | 119  (26%) | 208  (44%) | **468** | .183  (*3.4*) |
| Total medication tasks | 207  (23%) | 197  (22%) | 489  (55%) | **893** | **≤.001**  (*29.0*) |

**Table 8 Frequency of ‘Investigations and Procedures’ tasks observed by role**

|  | DR  n (%) | NP  n (%) | RN  n (%) | Total  n (%) | p (*χ^2^*) |
| --- | --- | --- | --- | --- | --- |
| Order  Investigation/ Procedure | 46  (44%) | 35  (33%) | 24  (23%) | 105  (100%) | **≤.001**  (*24.6*) |
| Prepare Investigation/Procedure | 70  (19%) | 119  (32%) | 178  (49%) | 367  (100%) | **.002**  (*12.3*) |
| Perform Investigation/Procedure | 30  (17%) | 73  (43%) | 68  (40%) | 171  (100%) | **≤.001**  (*20.5*) |
| Total | 146  (23%) | 227  (35%) | 270  (42%) | 643  (100%) | **≤.001**  (*20.1*) |

Pearson χ^2^, zero cells have an expected value <5, significant results bolded at p<.05

**Table 9 Count of investigation-related procedures performed, recorded in task details spreadsheet**

|  | DR  n | NP  n | RN  n | Total  n |
| --- | --- | --- | --- | --- |
| Venepuncture | 10 | 9 | 17 | **36** |
| Urinary Analysis | 1 | 1 | 10 | **12** |
| Electrocardiograph |  |  | 10 | **10** |
| Blood Sugar |  | 2 | 3 | **5** |
| Spirometry |  |  | 2 | **2** |
| Bladder Scan |  | 1 | 1 | **2** |
| Ultrasound | 2 |  |  | **2** |
| Blood Gas |  |  | 1 | **1** |
| **Total** | **13** | **13** | **44** | **70** |
